# Filament Advance Detection Sensor for Fused Deposition Modelling 3D Printers

**DOI:** 10.3390/s18051495

**Published:** 2018-05-09

**Authors:** Enrique Soriano Heras, Fernando Blaya Haro, José M. de Agustín del Burgo, Manuel Islán Marcos, Roberto D’Amato

**Affiliations:** 1 Departamento de Ingeniería Mecánica, Universidad Carlos III de Madrid, Avda. de la Universidad, 30, 28911 Leganés, 28012 Madrid, Spain; 2 Escuela Técnica Superior de Ingeniería y Diseño Industrial, Universidad Politécnica de Madrid, Ronda de Valencia, 3, 28012 Madrid, Spain; fernando.blaya@upm.es (F.B.H.); jm.deagustin@alumnos.upm.es (J.M.d.A.d.B.); manuel.islan.marcos@upm.es (M.I.M.); r.damato@upm.es (R.D.A)

**Keywords:** rapid prototyping, fused deposition, filament jams, extrusion failures, photogrammetry, manufacturing system

## Abstract

The main purpose of this paper is to present a system to detect extrusion failures in fused deposition modelling (FDM) 3D printers by sensing that the filament is moving forward properly. After several years using these kind of machines, authors detected that there is not any system to detect the main problem in FDM machines. Authors thought in different sensors and used the weighted objectives method, one of the most common evaluation methods, for comparing design concepts based on an overall value per design concept. Taking into account the obtained scores of each specification, the best choice for this work is the optical encoder. Once the sensor is chosen, it is necessary to design de part where it will be installed without interfering with the normal function of the machine. To do it, photogrammetry scanning methodology was employed. The developed device perfectly detects the advance of the filament without affecting the normal operation of the machine. Also, it is achieved the primary objective of the system, avoiding loss of material, energy, and mechanical wear, keeping the premise of making a low-cost product that does not significantly increase the cost of the machine. This development has made it possible to use the printer with remains of coil filaments, which were not spent because they were not sufficient to complete an impression. Also, printing models in two colours with only one extruder has been enabled by this development.

## 1. Introduction

The application of 3D printing technologies is ideal not only for the home-user or for tooling production, but the improvements of the quality and of the mechanical property mean that they are increasingly being used for direct manufacturing [1]. In fact, for aerospace industry—which needs to produce a small number of highly complex aircraft components—the application of 3D printing technologies is ideal [2,3]. In the medical sector, in the same manners, due to the need for personalized one-off products, the AM is the ideal technique to address this need. This is can bee seen in several applications like orthodontics, prosthetics, orthotics, implants, and organ replacement [4,5].

Nowadays, the use of 3D printers has extended beyond the research laboratories. It is possible to find them, more and more frequently, in houses, where they are used by non-technical users; or in factories, where an error in the operation can suppose great losses [6]. Therefore, these machines must remain 100% reliable with near-zero failed prints due to mechanical and electro-mechanical malfunctions.

After several years of development and improvement, the most important failure has not been corrected, jams in the extruder. Some researches and engineers have optimized the grip force on the 3D printer filament and even have developed novel feeding mechanisms [7,8]. This extrusion problem occurs to FDM 3D printers when the filament does not move as it is desired, and may be due to damage, stress, dust, and small debris in filament. Nevertheless, the most common problems arise from a wrong filament diameter, a breaking of the filament, or simply that the filament coil is over. In these cases, the printer keeps on moving, but it does not deposit any material [9].

**Figure 1:**
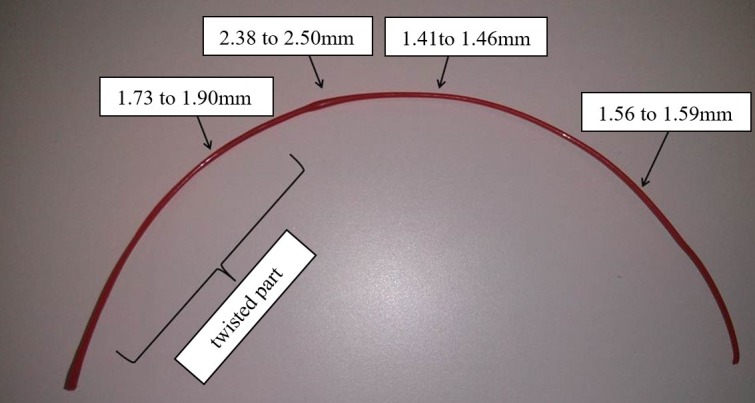
Defects in a filament sample.

Although manufacturers and researches are constantly improving polymer manufacturing processes, including fiber spinning and injection molding, the product quality and production efficiency is influenced by multiple processing and material parameters—such as the nominal shear and shear history, process temperature, or long chain branching—mechanisms that currently are not completely understood. The control and optimization of such operations contribute to get closer and closer to the nominal filament size but it still moves in fairly large tolerances [8,10,11,12]. In Figure 1, it is possible to see the imperfections that can be found in a new filament.

The aim of this study is to present a new method in order to detect all these extrusion failures that can may be produced by: a coil knot, an extruder jamming or, simply, that the filament coil is over. This can detect the correct filament advancement in the extruder. To reach this goal, it is initially thought of a mechanical switch that detects when the filament fails to move, but although it seems trivial to cases in which the filament breaks or runs out, it is more difficult to detect the correct advance. For this reason, we propose to use a rotation encoder driven by the movement of the filament. The printer should consult repeatedly, while printing, that the encoder is rotating and therefore the filament goes forward. In the event that no progress is detected, the machine will stop and offer the option to change the filament, reload it, and continue printing without having to discard the part.

## 2. Review of Extruder-Filament Sensors Used for Current 3D Printers

### 2.1. Mechanical Sensor

Mechanical sensors have been widely implemented in 3D printers, majority of them use a mechanical button to stay on while filament is detected could easily detect filament end or breakage to stop the printing. It is possible to find some detection systems using mechanical filament breakage sensors, but this kind of system does not solve the main problem, which is a filament jam, due to the state of the switch being unable to change [13].

### 2.2. Load Cell Sensor

As the extruder feeds the filament to the hot end, the extruder is effectively pushing against the filament, causing the extruder to apply extra load on the load cell. Load cells have strain gauges attached that change in electrical resistance when under different loads. This resistance change provides small voltage levels that can be amplified and then read by an analogue to digital converter. Unfortunately, a load cell sensor could make it difficult to calibrate without a suitable weighing platform and stand [14].

### 2.3. Rotary Encoder

A rotary encoder, is an electro-mechanical device that converts the angular position or motion of a shaft or axle to an analog or digital code [15]. There are two main types: absolute and incremental. The output of absolute encoders indicates the current position of the shaft, making them angle transducers. The output of incremental encoders provides information about the motion of the shaft, which is typically further processed elsewhere into information such as speed, distance, and position. The encoder may have mechanical problems due to the high accuracy needed in fabrication. Environmental pollution can be a source of interference in optical transmission as well, which is particularly sensitive to shock and vibration. The rotary encoders’ operating temperature is limited by the presence of electronic components.

#### 2.3.1. Mechanical Encoder

Mechanical encoders have an axis that spins internally, thus activating different pins depending on the direction of rotation and speed. Although this type of encoder seems easy to use as first, the resistance of the rotation axis is not desired. It could increase the resistance of the filament feed and it could affect the proper operation of the extruder.

#### 2.3.2. Optical Encoder

The principle of operation of an optical encoder is based on the so-called photo couplers. These are small chips consisting of a diode as a photo emitter and a transistor which act as photoreceptors. This element is responsible for detecting the presence/absence of light through a concentric axis, it is manufactured with slots that allow the light to go through the disc to obtain the final measure [16].

## 3. Filament Auto-Detection System Development

### 3.1. Election of the Sensor

The weighted objectives method is one of the most common evaluation methods for comparing design concepts based on an overall value per design concept [17]. This method makes a direct comparison of the design. To do this, the weighted objective method assigns scores to the degree to which a design alternative satisfies a criterion. However, the criteria that are used to evaluate the design alternatives might differ in their importance. For example, the cost can be of less importance than appealing aesthetics. To deal with it, the method allows the assigning of weights to different criteria. This allows the decision-maker to take into account the difference in importance between criteria. At the end of the method, the alternative that best suits the criteria can be seen.

The biggest disadvantage of using other methods like the Datum method or the Harris profile is that the scores per criterion cannot be aggregated into an overall score of the design alternative.

The selected criteria, compared in Table 1, are the following:

E1. Filament detection (yes-no)
E2. Detecting the advance of the filament
E3. Not interference with normal movement of the filament
E4. Adaptability of the output signal
E5. Price
E6. Durability

Taking into account the scores (see Table 2), and as expected, the sensor that best meets the specifications is the optical encoder. In this work, an inexpensive bi-directional optical incremental encoder is used.

### 3.2. Hardware Assembly

#### 3.2.1. Assembly Part Design

Once the sensor is chosen, it is necessary to design the part where it will be installed. It must be taken into account that it cannot interfere with the normal function of the machine. For the 3D printing of the prototype, a Prusa i3 BQ Hephestos printer has been used. In order to do it, we will employ photogrammetry scanning methodology since it will be possible to do it in a precise way [18].

This method uses reverse engineering, thus allowing us to reduce the costs of the development.

After taking numerous pictures of the object, they are processed using a computer software so that common points are identified on each image. A line of sight (or ray) can be constructed from the camera location to the point on the object. It is the intersection of these rays (triangulation) which determine the three-dimensional location of the point.

**Figure 2:**
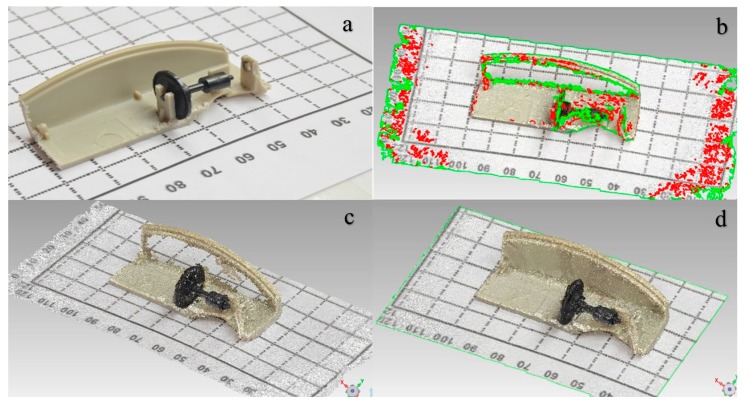
Images for photogrammetry process: (**a**) Real figure; (**b**) point cloud; (**c**) and (**d**) digital model.

The result of the process is a digital tridimensional object which can be used as a model to design the rest of the parts. It is interesting to include graphic scales to get the correct dimensions of the digital model. Figure 2a shows a sample of a total of 74 images involved in the process.

After taking numerous pictures of the object, they are processed using computer software so that common points are identified on each image.

A line of sight (or ray) can be constructed from the camera location to the point on the object. It is the intersection of these rays (triangulation) that/which determines the three-dimensional location of the point.

The result of the process is a digital tridimensional object which can be used as a model to design the rest of the parts. It is interesting to include graphic scales to get the correct dimensions of the digital model.

The software locates the pictures and shapes a point cloud of the scanned object. The process is shown in Figure 2a (real figure), b (point cloud), c,d (digital model) where it is possible to see the pictures completely orientated and the formatted points cloud.

As it is possible to notice in Figure 2c, there are some defective parts. This is due to the brightness of the object, so it is necessary to perform a repair of the digital model, so a model as similar as possible to the original can be reached.

In order to achieve this, first of all a filter of the points is performed to remove the noise by eliminating points spaced of the set a specified size. After this, different holes are detected. In this case, a total of 698 of holes which 670 are closed automatically since have a small size. The remaining 28 holes are manually closed to keep the original form. An automatic reparation of errors is carried out, and finally, it is possible to get the solid digital model. Figure 2d shows an image of the final virtual model.

Once the three-dimensional solid model is obtained, it is exported to a 3D design program for modeling the part where it will be assembled.

**Figure 3:**
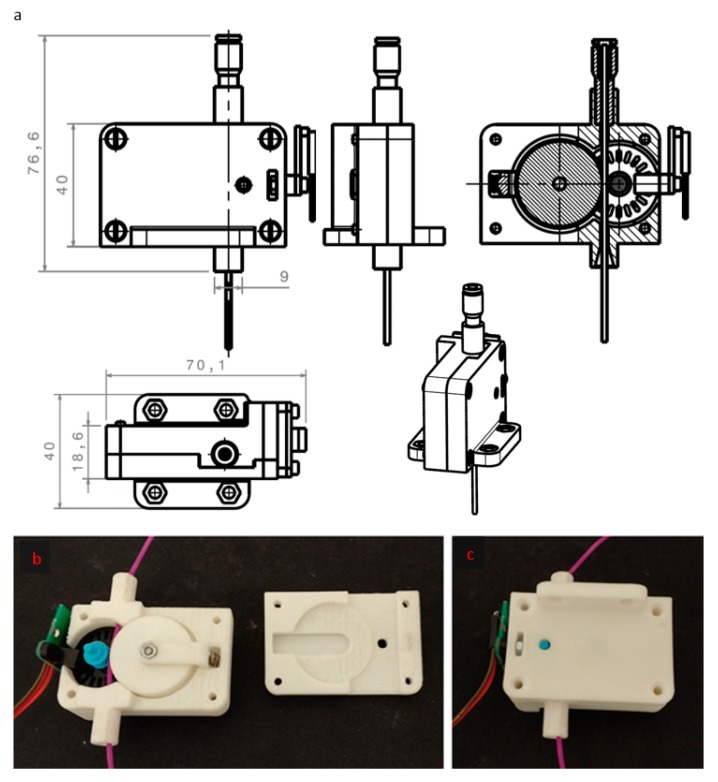
Real product: (**a**) Optimal design model; (**b**) and (**c**) Real product made with a 3D printer.

In this way, it is possible avoid design errors that have to make too many iterations to find the optimal model. In Figure 3b,c, the real product made with a 3D printer is showed disassembled and assembled. As it is possible to see, the filament will make that the encoder moves when it is advancing.

#### 3.2.2. System Assembly

The whole system is installed on the top of the printer, so that the filament goes through the sensor. After the sensor, the filament is leaded through a Teflon tube to the hot-end, analogously to the Bowden system.

After checking that the system does not interfere in the normal function of the machine, it is connected to the main electronic board of the printer (an Arduino Mega board).

#### 3.2.3. Firmware Modifications

Once the system is installed, it is necessary to modify the firmware of the machine, so it is possible to get the sensor signal and act accordingly. Since the encoder works asynchronously, it is necessary to use program interruptions to get the signal correctly. These interruptions will detect whether the filament is moving or is blocked. Moreover, it will be possible to calculate the speed at which the filament is advancing in order to be sure about the quantity of material deposited.

However, after repeated tests, it is observed that the interruptions take place very frequently, which interferes with the operation of the printer. To avoid this problem, a new electronic configuration is proposed. A slave board will be programmed to control the encoder signal and to communicate with the master board to indicate if there is an error in the filament advance. This way, we can dedicate this new board to also calculate the speed of the filament, as with only one board it was very difficult due to the main boar must work in a fluent way to control the motors correctly. A new pause menu is also implemented in the master board, since error filament was not previously available.

Once an error in advancing filament is detected, the printer activates the implemented pause mode due to filament error, at which point it is possible to load and unload the actual filament to continue printing to avoid losing the piece.

## 4. Performance Evaluations

### 4.1. Filament Defects

Although most of filament producers for 3D machines are constantly developing and improving their products, the manufacturing method has so far prevented achieving a filament with a constant diameter. This excess in diameter is sometimes too much for the machine, causing bad finishing models, jamming of the extruder, or even damaging the extruder. In order to check the filament diameter of different producers in this study, a sample with a length of 300 cm, for each providers, was taken. The filament diameters was investigate by using a sensor with a resolution of 0.01 mm.

**Figure 4:**
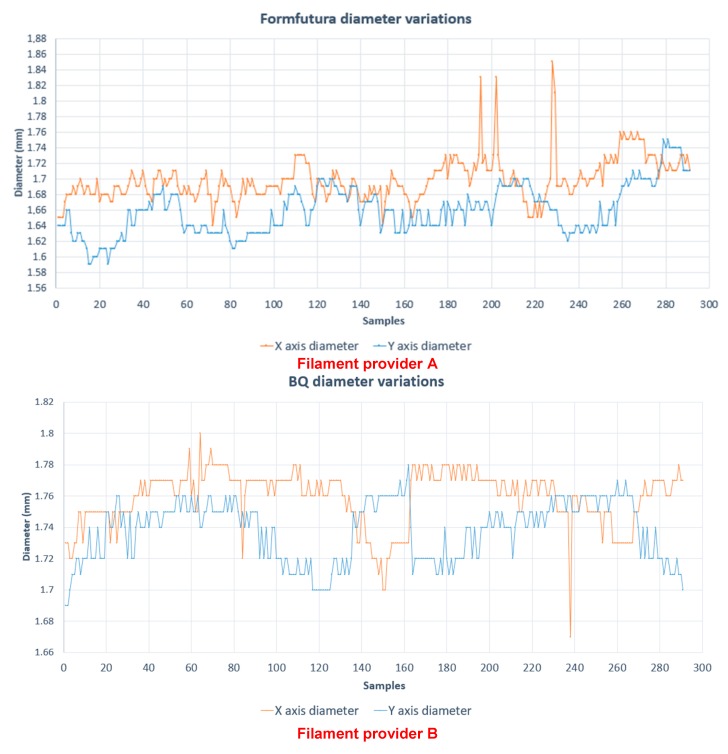

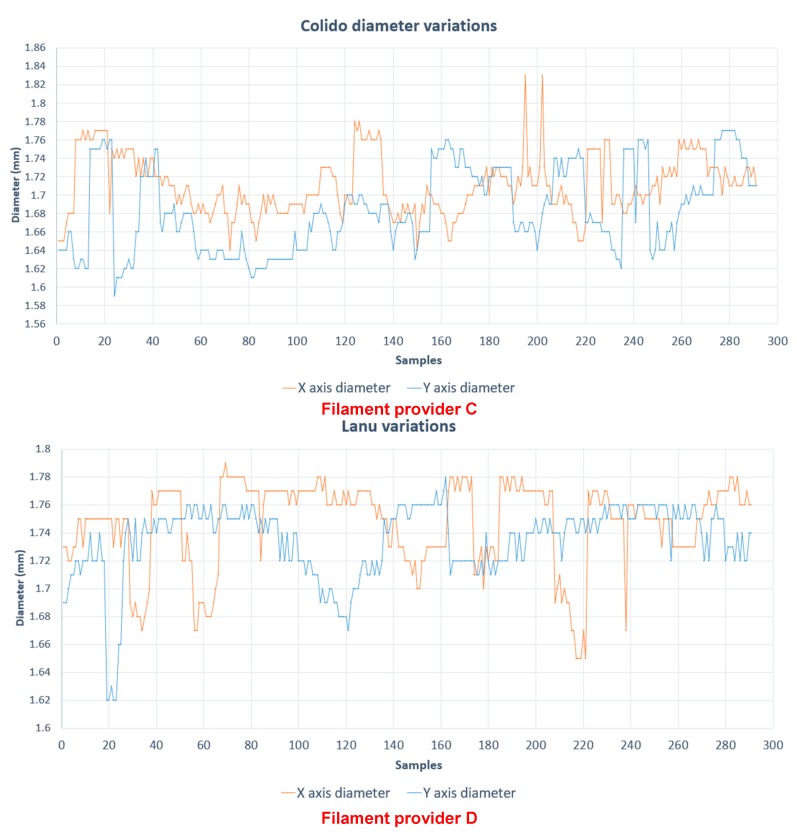
Diameter variation for four different filament providers (A, B, C, D).

Figure 4 shows the diameter variation (whose nominal value is 1.75 mm) in *X* and *Y* axis, from different filament providers (A, B, C, D).

By analyzing these samples (see Table 3), it is possible to see that the diameter varies from 1.56 mm to 1.85 mm. This is the principal reason for extruder obstructions.

Any of these failures means that leaving the printer in operation will mean losing the piece that was being created, requiring the manufacturer to start again. In addition, by continuing printing without really extruded plastic, the machine consumes energy and produces an unnecessary wastage. Due to this reason, the operator must be aware of the machine as long as it is operating, ensuring that the plastic flows without problem, which is especially difficult when the piece takes several hours to be produced.

### 4.2. Evaluation of the Implemented System

The installed system does not affect the print quality of the machine. It has been found that the time set for detecting advancing filament problems detects an error in time without producing false positives. In Table 4, the error is displayed on the printer.

**Figure 5:**
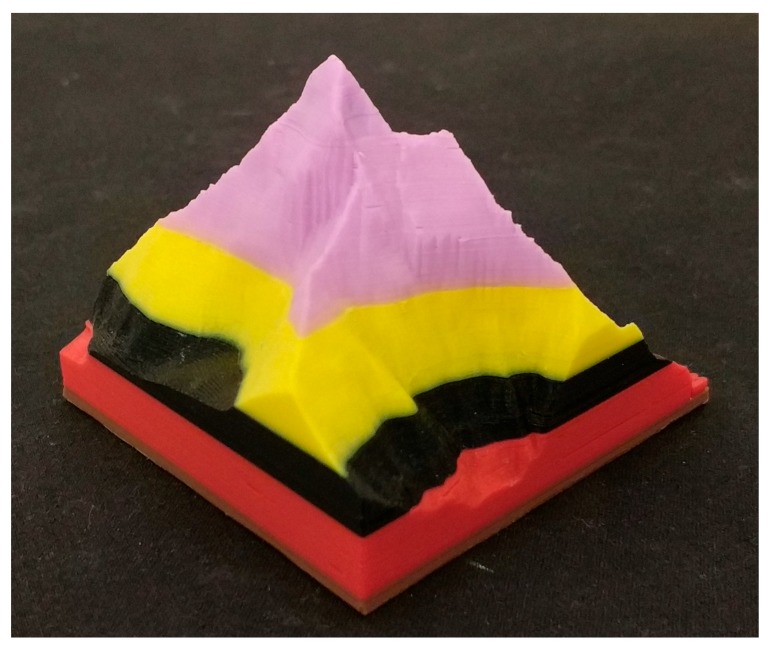
Model produced after five filament advance problems.

Figure 5 shows an object produced by a 3D printer where two filament feed errors were forced.

## 5. Conclusions

The aim of the paper is to present a system to detect extrusion failures in fused deposition modelling (FDM) 3D printers by sensing that the filament is moving forward properly. In this study, different sensors and the weighted objectives method, one of the most common evaluation methods for comparing design concepts based on an overall value per design concept were used.

The installed system achieves perfectly detect the advance of the filament without affecting the normal operation of the machine. The implementation of a slave board that gets the encoder signal, can calculate the filament speed so that it is possible not only to know when there is a filament advance problem, but also to adjust the extrusion speed if the adjusted speed does not match with the real speed.

This has enabled the use of the printer with remaining coil filament which had not been used before because it was insufficient to complete an impression. With this system, when the filament finishes, the printer enters into a standby state waiting for the user to introduce a new filament.

Therefore, the primary objective of the system is achieved, avoiding loss of material, energy, and mechanical wear, keeping the premise of making a low-cost product that does not significantly increase the cost of the machine. The electronic encoder has a price of 2–5 €, the box for it has be produced with a 3D printer and the new electronic slave board has a price between 1–3 €.

This low cost system could be implemented in any printer, as it does not interfere with the normal functions of the machine, as it is mounted at the entrance of the filament.

## Figures and Tables

**Table 1:** Filament detection sensor evaluation.

Sensor	E1	E2	E3	E4	E5	E6	Amount	Compensation	Weight	%
**E1**	X	0.0	0,0	1.0	0.5	0.5	1.5	2.5	0.167	16.67
**E2**	1.0	X	0.5	0.5	0.5	0.5	2.5	3.5	0.233	23.33
**E3**	1.0	0.5	X	1.0	0.5	1.0	3.0	4.0	0.267	26.67
**E4**	0.0	0.5	0.0	X	0.5	0.0	1.0	2.0	0.133	13.33
**E5**	0.5	0.5	0.5	0.0	X	0.5	1.5	2.5	0.167	16.67
**E6**	0.5	0.5	0.0	1.0	0.5	X	2.5	3.5	0.233	23.33
**Total**							9.5	14.5	0.967	96.67

**Table 2:** Sensor marks.

Sensor 1	Mark	Satisfaction	Final Mark	Sensor 2	Mark	Satisfaction	Final Mark	Sensor 3	Mark	Satisfaction	Final Mark
E1	16.67	100%	16.67	E1	16.67	100%	16.67	E1	16.67	100%	16.67
E2	23.33	0%	0.00	E2	23.33	100%	23.33	E2	23.33	100%	23.33
E3	26.67	100%	26.67	E3	26.67	25%	6.67	E3	26.67	100%	26.67
E4	13.33	100%	13.33	E4	13.33	75%	10.00	E4	13.33	75%	10.00
E5	16.67	100%	16.67	E5	13.33	75%	10.00	E5	13.33	75%	10.00
E6	23.33	75%	17.50	E6	16.67	75%	12.50	E6	16.67	50%	8.33
		Total	73.33			Total	79.17			Total	95.00

**Table 3:** Maximum, minimum, and mean of filaments.

Provider	Maximum (mm)	Minimum (mm)	Mean (mm)
A	1.85	1.59	1.68
B	1.8	1.67	1.75
C	1.84	1.56	1.75
D	1.83	1.59	1.78

**Table 4:** Error menu.

Filament error. Push the button to change the filament.
Extract the filament when the motor stopes.
Insert the new filament and push the button.
When you see come out the filament, press the button to continue printing.
